# To the Editors of the Medical and Physical Journal

**Published:** 1803-07-01

**Authors:** Thomas Beddoes

**Affiliations:** Clifton


### *To the Editors of the Medical and Physical Journal.*

GENTLEMEN,

PARTICULAR circumstances having induced me to make a very extensive enquiry concerning the progress and nature of the Influenza, I have before me a great mass of opinions and facts, with permission to make them public. Your Journal would, I think, do infinitely more justice to my correspondents than a separate publication, and if you can spare them and me room, I will place the returns to my queries in order, and add a commentary. The whole will make three communications sufficiently long, of which the first may appear in your next number.*

*Clifton,*
*June* 17, 1803.

I am, &c.

THOMAS BEDDOES.

---

### *To the Editors of the Medical and Physical Journal.*

* The Editors much approve of Dr. Beddoes's proposed mode of publishing his communications on the late Epidemic, and they will certainly accommodate him in the best manner in their power, if the manuscript arrives in time.

